# Allergic diseases of the skin and drug allergies – 2036. Exanthematous drug eruption to prasugrel: a case report

**DOI:** 10.1186/1939-4551-6-S1-P121

**Published:** 2013-04-23

**Authors:** Praveen Buddiga, Muhammad Hassan Bashir, Malik Baz

**Affiliations:** 1 University of California, San Francisco, Baz Allergy, Asthma & Sinus Center, Fresno, CA, USA; 2 Baz Allergy, Asthma and Sinus Center, Fresno, CA, USA

## Introduction

Prasugrel is a thienopyridine anti-platelet agent used for managing cardiac patients under going percutaneous coronary intervention (PCI) or stent placement to reduce thrombotic event rate. Prasugrel has multiple side effects, however, only one case of an allergic reaction has been reported to the drug before.

## Methods

A 54 year old male with past medical history of diabetes, hypertension and hyperlipidemia presented with angina. The cardiac catheterization showed significant coronary artery disease with left anterior descending artery blockage. During the process of placing 4 drug eluting stents into the artery a loading dose of Prasugrel 60 mg was administered and subsequently a Prasugrel 10 mg dose was given on daily basis until day 7. Per the patient he started having some itching on the back of his neck and face immediately after taking the loading dose of Prasugrel. Patient continued the Prasugrel for 7 more days before he developed a pruritic maculopapular rash starting on his extremities and then spreading to the trunk and back. Patient sought an allergist consult immediately after the development of this rash. The allergist’s assessment was that this was a drug eruption of morbilliform nature. Patient was started on a oral steroid - Prednisone 40 mg PO daily tapering to 10 mg over 7 days with antihistamines (diphenhydramine 50 mg at night + cetirizine 10 mg in the morning). Prasugrel was discontinued and the patient was started on clopidogrel 10 mg as an alternative for antithrombotic prophylaxis. Patient’s rash slowly dissipated over one week. The patient has not developed the rash again on the alternative regimen of clopidogrel.

## Conclusion

Exanthematous drug eruptions may occur in up to 1-5 % of first time users of most drugs. The drug eruption typically appears 4-21 days after the person starts taking the causative agent. The eruption is usually characterized by symmetrically distributed macules and papules that may coalesce. Prompt recognition to the signs and symptoms of a cutaneous reaction secondary to a drug should alert the clinician to act by stopping the causative drug and using acceptable alternatives.

